# Glitches in the brain: the dangerous relationship between radiotherapy and brain fog

**DOI:** 10.3389/fncel.2024.1328361

**Published:** 2024-03-07

**Authors:** Noemi Marino, Martina Bedeschi, Melania Elettra Vaccari, Marco Cambiaghi, Anna Tesei

**Affiliations:** ^1^Bioscience Laboratory, IRCCS Istituto Romagnolo per lo Studio dei Tumori (IRST) “Dino Amadori”, Meldola, Italy; ^2^Department of Neurosciences, Biomedicine and Movement Sciences, University of Verona, Verona, Italy

**Keywords:** brain fog, cognitive impairment, neuroinflammation, brain tumors, sigma receptors, P2X7 receptors, transcranial direct current stimulation, hyperbaric oxygen

## Abstract

Up to approximately 70% of cancer survivors report persistent deficits in memory, attention, speed of information processing, multi-tasking, and mental health functioning, a series of symptoms known as “brain fog.” The severity and duration of such effects can vary depending on age, cancer type, and treatment regimens. In particular, every year, hundreds of thousands of patients worldwide undergo radiotherapy (RT) for primary brain tumors and brain metastases originating from extracranial tumors. Besides its potential benefits in the control of tumor progression, recent studies indicate that RT reprograms the brain tumor microenvironment inducing increased activation of microglia and astrocytes and a consequent general condition of neuroinflammation that in case it becomes chronic could lead to a cognitive decline. Furthermore, radiation can induce endothelium reticulum (ER) stress directly or indirectly by generating reactive oxygen species (ROS) activating compensatory survival signaling pathways in the RT-surviving fraction of healthy neuronal and glial cells. In particular, the anomalous accumulation of misfolding proteins in neuronal cells exposed to radiation as a consequence of excessive activation of unfolded protein response (UPR) could pave the way to neurodegenerative disorders. Moreover, exposure of cells to ionizing radiation was also shown to affect the normal proteasome activity, slowing the degradation rate of misfolded proteins, and further exacerbating ER-stress conditions. This compromises several neuronal functions, with neuronal accumulation of ubiquitinated proteins with a consequent switch from proteasome to immunoproteasome that increases neuroinflammation, a crucial risk factor for neurodegeneration. The etiology of brain fog remains elusive and can arise not only during treatment but can also persist for an extended period after the end of RT. In this review, we will focus on the molecular pathways triggered by radiation therapy affecting cognitive functions and potentially at the origin of so-called “brain fog” symptomatology, with the aim to define novel therapeutic strategies to preserve healthy brain tissue from cognitive decline.

## 1 Introduction

Cancer-related cognitive deficits (CRCDs), also known as “brain fog,” are common among cancer patients (Hardy et al., 2023) and include difficulties in short-term and working memory, attention, processing speed, verbal fluency, and executive function (Jean-Pierre et al., 2012; McDougall et al., 2014; Prasad et al., 2015). Among the potential reasons for brain fog aside from psychological distress are chemotherapy-or radiation-related neurotoxicity (Hardy et al., 2023). However, the etiology of CRCDs remain still elusive as they arise during treatment but can also persist for an extended period after the end of chemo-or radio-treatment (Falleti et al., 2005; Eberhardt et al., 2006; Ahles et al., 2012).

Brain tumors are one of the most aggressive and detrimental forms of cancer. In particular, for brain tumors and for brain metastasis originating from extracranial tumors, the standard treatment includes surgery, chemotherapy and radiation. Glioblastoma multiforme (GBM) represents the most common and aggressive brain tumor in the aging population and accounts for 58% of all gliomas in the elderly. The prognosis for patients with GBM remains dismal, with overall survival of 12–18 months. Notwithstanding, though GBM is a rare tumor with a global incidence of less than 10 per 100,000 people, its poor prognosis makes it a crucial public health issue. Early diagnosis and the type of treatment chosen do not affect GBM patient survival rate, making screening programs unhelpful. Long-term survival in GBM, defined as survival beyond 3 years, remains scarce, with estimates ranging from 3 to 5%. While extensive research is underway to develop novel therapies for extending survival, the impact of tumor and treatment on the cognitive status of survivors remains relatively understudied.

The current standard of care for Glioblastoma multiforme is the Stupp’s protocol, developed in 2005, which involves a two-stage approach: debulking surgery followed by a combination of radiotherapy (RT) and chemotherapy (Stupp et al., 2005). RT is the mainstay treatment (Marsh et al., 2010; McDuff et al., 2013; Owonikoko et al., 2014) due to its capability to uniformly penetrate both the brain and tumor parenchyma, overcoming resistant cells (McDuff et al., 2013). However, on the other side of the coin, cranial radiation may induce a cognitive decline, the most common radio-correlated neurotoxic effect at any patient’s age observed also as a result of doses much lower than those that can cause radionecrosis (Makale et al., 2017).

Radiation therapy, an integral component of modern cancer treatment, holds particular significance for primary brain tumors. Often the sole modality that offers substantial survival and quality-of-life benefits, RT plays a crucial role in the management of these malignancies.

Over the past few decades, radiotherapy for brain tumors has undergone significant technological advancements across all aspects of treatment, including patient immobilization, imaging, treatment planning, and precise delivery. This includes better imaging, planning, and delivery methods. These advancements, especially in imaging and radiation technology, allow for more precise targeting of tumors and less damage to healthy brain tissue (Scaringi et al., 2018). In particular, the intensity-modulated radiation therapy (IMRT) and volumetric modulated arc therapy (VMAT) “shape” the radiation beams to closely fit the tumor’s unique shape, minimizing harm to healthy tissue and reducing side effects while maximizing the treatment’s effectiveness (Scaringi et al., 2018; Kotecha et al., 2021). Stereotactic irradiation represents an advanced iteration of conventional external beam radiation therapy (CRT). It uses special headgear to hold patients perfectly still, allowing incredibly precise targeting of the tumor with submillimeter accuracy. This reduces the amount of healthy brain tissue exposed to radiation, potentially lowering the risk of long-term side effects. The treatment can be given in one or multiple sessions, still delivering high doses to the tumor. Another relatively new technique is FLASH RT, defined as a single ultra-high dose-rate RT (higher than 40Gy/S), based on the proton’s capacity of deliver little energy with the highest energy release in the target volume, leading no dose leakage and reducing damage on healthy tissue (Hughes and Parsons, 2020; Huang and Mendonca, 2021; Lin et al., 2022).

RT has established itself as one of the three mainstays of GBM treatment, alongside surgery and chemotherapy (Orth et al., 2014). Beyond its direct and indirect DNA damage-induced local control of target lesions in cancer cells, recent preclinical and clinical evidence suggests that RT may also modulate antitumor immune responses by inducing immunogenic cell death and reconfiguring the tumor microenvironment (TME). In particular, GBM is characterized by high inter-and intra-tumor heterogeneity and a very complex TME, composed not only of neoplastic cells but also of nervous cells (i.e., astrocytes and neurons), stem cells, fibroblasts, vascular as well as varieties of host and infiltrating immune cells. This has led many to evaluate RT as a partner therapy to immuno-oncology treatments, a research field very relevant in brain tumors, where the blood–brain barrier (BBB) significantly limits the penetration of antineoplastic drugs into the brain and consequently the achievement of therapeutic sufficiently high concentrations. While RT offers potential benefits in treating brain tumors, it is also associated with a common complication: cognitive decline. Despite the prevalence of this issue, the underlying mechanisms responsible for this dysfunction remain largely unclear. Consequently, there are currently no effective preventive measures or treatments available.

This review aims to shed light on this critical yet understudied issue.

## 2 The relationship between RT and brain fog

### 2.1 RT and neuroinflammation: mastering the duality of a double-edged blade

Our current understanding of the mechanisms underlying radiation-induced brain injury centers on the immediate depletion of neural stem cells and the subsequent disruption of hippocampus-mediated functions, including learning and memory. Indeed, different studies have documented that stress leads to a reduction of dendritic, spine, and synaptic material in the hippocampus and prefrontal cortex (Wager-Smith and Markou, 2011). Additionally, a single 10-min session of swim stress has been shown to cause dendritic length loss in the infralimbic cortex (Izquierdo et al., 2006). In the context of RT effects and cancer, it is well known radiation ability to induce an “immunogenic hub” of great relevance for the local (bystander effect) and remote (abscopal effect) antitumor effects, as described for several solid tumors by different groups (Formenti and Demaria, 2013; Baskar et al., 2014; Klammer et al., 2015; Marín et al., 2015). However, RT could have a hidden side in the brain. Indeed, in addition to its direct cytotoxic effect on neuronal cells, RT may negatively impact on the cells of TME directly or by inducing the release of inflammatory mediators such as adenosine triphosphate (eATP), interferons, and chemokines to the extracellular space (Greene-Schloesser et al., 2012; Herrera et al., 2017) potentiating glioma cell growth and invasion or contributing to build up an immunosuppressive milieu (Wang and Haffty, 2018).

The same glioma cells are known to increase oxidative stress and stimulate the release of immunosuppressive molecules such as interleukin-6 (IL-6), IL-10 and tumor growth factor beta (TGF-β), which in turn reprogram the immune components of TME such as microglia to a pro-tumorigenic phenotype (Alghamri et al., 2021). This condition can lead to the loss of BBB integrity, exposing the brain to adverse substances from the periphery, and to host immune cells, that can disrupt the homeostasis of the CNS (Alghamri et al., 2021). In support of this, the impact of myeloid cells on TME is compared by Buonfiglioli and Hambardzumyan to “the mythological evil three-headed dog, Cerberus,” that guards the underworld as well as microglia cells play a triple protecting and supporting role on tumor. In fact, these myeloid cells promote tumor growth, modulate immune suppression, and exacerbate cerebral edema (Buonfiglioli and Hambardzumyan, 2021).

Furthermore, several works showed that irradiated microglia may induce astrogliosis, release of neurotoxic factors, compromising the BBB integrity with consequent immune cells invasion and neuronal cell death (Hwang et al., 2006; Wilson et al., 2009; Liddelow et al., 2017). The induction of a reactive state in microglia following cranial irradiation treatment have been shown to be associated with deficits in neural precursor, neuronal cell population maintenance and neurogenesis, in synaptic structure and function, and myelin plasticity. During development and under normal physiological conditions, microglia play a crucial role in shaping neural circuit refinement by eliminating excess dendritic and synaptic connections (Stevens et al., 2007; Schafer et al., 2012). Moreover, these cells exhibit complex branching patterns and display remarkable mobility in response to injury or disease, rapidly migrating to the affected area to engulf cellular debris. Activated microglia are also observed in various neurodegenerative disorders, such as Alzheimer’s disease (AD) (Hong et al., 2016) and Parkinson’s disease (PD) (Lecours et al., 2018), where microglial activation contributed to aberrantly increased synaptic pruning. Notably, microglia and astrocytes are overexpressed in the brain until 140 days post-irradiation in rats (Desmarais et al., 2015). In addition, microglial cells were found in an activated status with a classic amoeboid phenotype, harboring few ramifications and increased body volume in the area near the irradiation focus, while showing a steady state morphology with extended processes in the distal area (Constanzo et al., 2020). It is also known that microglia exhibit remarkable plasticity, adopting a spectrum of activation states ranging from fully inflamed, characterized by the release of pro-inflammatory cytokines, to alternatively activated, distinguished by the secretion of anti-inflammatory cytokines or neurotrophins. Consequently, microglia can transit from a homeostatic, neurotrophic state to a neurotoxic state (Luo and Chen, 2012). *In vivo* experiments on rats and mice demonstrated a dose-dependent reduction in hippocampal neurogenesis following ionizing irradiation, with higher radiation doses resulting in more pronounced impairments in both proliferating precursor cells and newly formed neurons (Tada et al., 1999). The deficit in neurogenesis is due mainly to radiation-induced perturbations in the neurogenic niche, rather than cell-intrinsic effects on the precursor cells (Monje et al., 2002, 2003). The IL-6-mediated inhibition of neuronal differentiation caused by radiation-activated microglia was postulated to be the central element in this microenvironmental disruption (Monje et al., 2002). In preclinical models, the direct contribution of radiation-induced microglial inflammation to cranial irradiation-mediated memory impairments is strongly supported by the evidence that anti-inflammatory drugs targeting microglia or depletion of microglia (Monje et al., 2003) using CSF1R inhibitors (Acharya et al., 2016) restore hippocampal neurogenesis and enhance cognitive function following irradiation. Finally, a recent study employing a glioma mouse model underscores the significance of non-tumor factors in memory impairment following cranial irradiation. The study suggests that microglial activation triggered by radiation exposure plays a more prominent role in memory dysfunction than tumor growth itself (Feng et al., 2018). While the applicability of these findings to humans warrants further investigation, the specifics may differ across glioma subtypes.

Furthermore, irradiated brain tissues show pathologic features resembling aging-associated neurodegeneration, including reduced neurogenesis, chronic oxidative stress and inflammation (Mrak, 2009; Wang et al., 2014). In response to radiation exposure and subsequent DNA damage accumulation, cells can undergo various cell type-specific responses, one of which is cellular senescence (Eriksson and Stigbrand, 2010). Notably, senescent cells, despite their inability to replicate, may evade clearance and accumulate in tissues, persistently releasing inflammatory factors that contribute to tissue damage (Tripathi et al., 2021). Consequently, radiation-induced cellular senescence has emerged as a crucial mediator of tissue dysfunction, fueling chronic inflammation and exacerbating radiation-induced side effects. Moreover, a burgeoning body of research suggests that astrocyte senescence and astrocyte-derived neuroinflammation could be identified as potential contributors to radiation-induced brain injury. While astrocytes perform numerous neuroprotective functions, including the production of neurotrophic factors, they may also promote neurodegeneration in certain diseases, such as AD, which is thought to be associated with the induction of a senescence-associated secretory phenotype (SASP). In addition, animal models of radiation-induced brain injury have revealed the presence of hypertrophic astrocytes that persist for at least 12 months following radiation exposure (Suman et al., 2013; Turnquist et al., 2016). Notably, a significant proportion of these enlarged astrocytes exhibit senescence, a crucial pathological feature that likely extends to other brain disease processes. Following brain injury, astrocytes undergo proliferation as part of reactive astrogliosis, a process that can lead to replicative senescence (Pekny and Pekna, 2014; Herranz and Gil, 2018). Elevated secretion of the SASP cytokines, IL-6 and IL-1β has been observed in animal models of radiation-induced brain injury and is suspected to impede neurogenesis, thereby contributing to cognitive decline (Haveman et al., 1998; Monje et al., 2003; Rola et al., 2004; Lee et al., 2010; Yang et al., 2017). Therapeutic interventions that target and mitigate neuroinflammation using anti-inflammatory drugs have demonstrated enhanced neurogenesis in radiation-induced brain injury (Marmary et al., 2016). In fact, in animal models, IL-6 has been shown to exacerbate radiation-induced senescence, further emphasizing the crucial role of chronic neuroinflammation in promoting radiation-induced brain injury (Turnquist et al., 2019). It is also been reported that the inhibition of full-length p53 regulates p21, RAD51, and IL-6, each of which has been shown to be important in radiation-induced injury and neurotoxicity. The same study also provided compelling evidence suggesting that the p53 isoform ∆133p53 holds therapeutic potential in preventing astrocyte senescence and mitigating astrocyte-mediated neuroinflammation. In addition, astrocyte dysfunction, even in the absence of neuronal or other cellular damage, can lead to memory loss. These abundant brain cells (not by chance, some gliomas more closely resemble cells of the astrocyte lineage) do play crucial roles, while their contribution to neurocognitive disorders such as dementia remains incompletely understood. A recent work by Licht-Murava et al. (2023) showed that abnormal immune activity in astrocytes is sufficient to cause cognitive deficits in dementia. In particular, the authors found that patients with AD or frontotemporal dementia have aberrant accumulation of TAR-DNA binding protein-43 (TDP-43) in hippocampal astrocytes. In Alzheimer’s disease mouse models, inducing widespread or hippocampus-targeted TDP-43 accumulation in astrocytes resulted in progressive memory loss and localized alterations in antiviral gene expression. Furthermore, Disruptions in astrocytic TDP-43 function contribute to cognitive decline through abnormal chemokine-mediated signaling between astrocytes and neurons (Licht-Murava et al., 2023).

**Figure 1:**
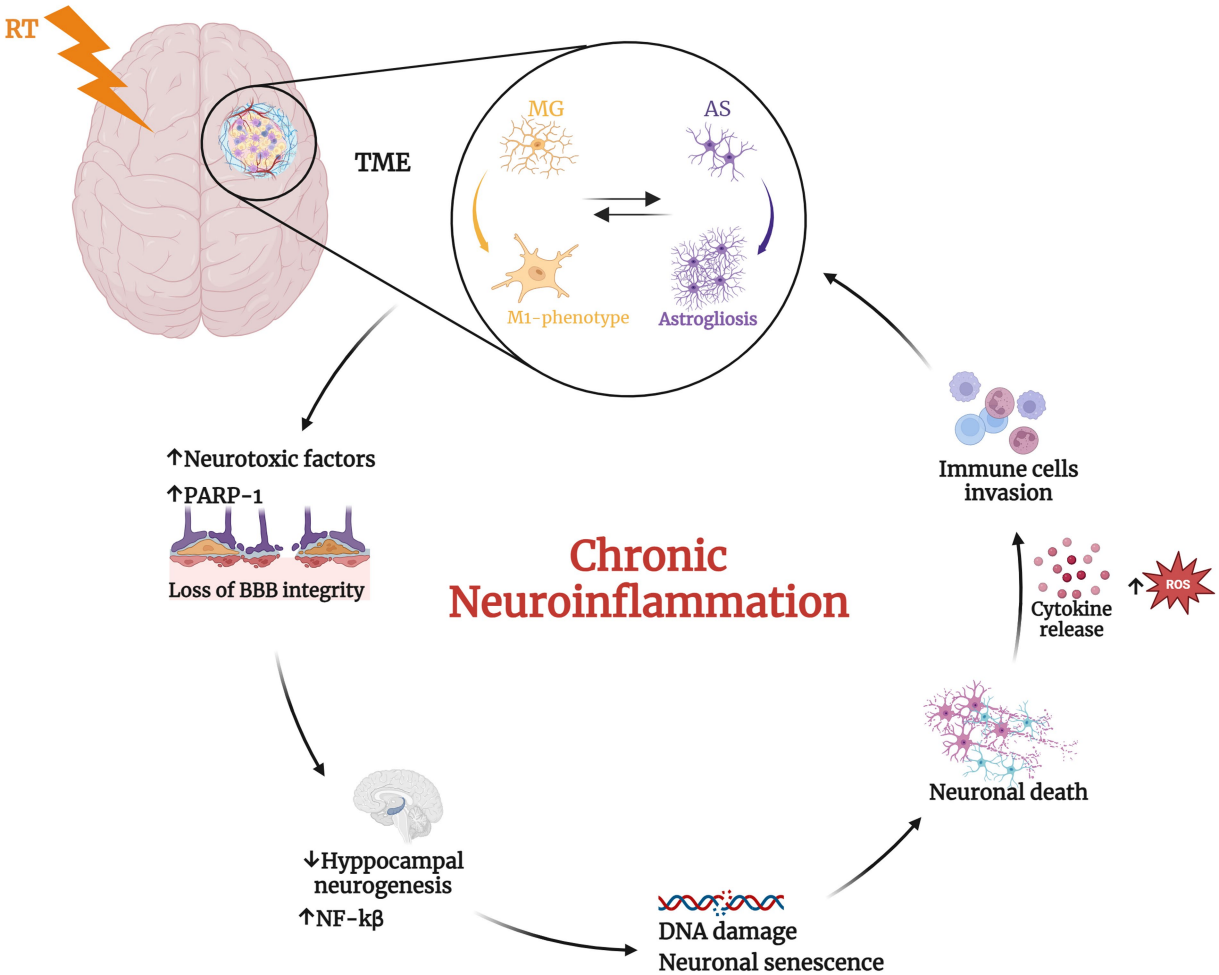
Radiotherapy (RT) induces significant changes in the tumor microenvironment. RT mediates the transition of microglial (MG) cells from a resting state to the activated pro-inflammatory M1-phenotype that induces prolonged activation of astrocytes (AS) leading to astrogliosis (Hwang et al., 2006; Wilson et al., 2009; Liddelow et al., 2017; Feng et al., 2018). This, in turn, promotes the phenotype switching of microglia in a vicious cycle. These phenomena determine neurotoxic factors release, the alteration of blood–brain barrier (BBB) integrity (Alghamri et al., 2021) and the upregulation of Poly ADP-ribose polymerase 1 (PARP-1), with consequent increase of nuclear factor kappa beta subunit (NF-kβ) and reduced neurogenesis (Ullrich et al., 2001; Chiarugi and Moskowitz, 2003; Kauppinen and Swanson, 2005; Kauppinen et al., 2011; Martínez-Zamudio and Ha, 2014; Stoica et al., 2014; Raghunatha et al., 2020; Gutierrez-Quintana et al., 2022). The consequent inflamed condition induces DNA damage, neuronal senescence, and ultimately neuronal death. These alterations further recall immune cells into the tumor microenvironment (TME) determining a chronic neuroinflammation status. Created with BioRender.com.

Finally, both *in vitro* and *in vivo* studies have demonstrated that the inflammatory response of microglia and astrocytes is mediated by PARP-1, with its activation triggering protein synthesis and proliferation (Gutierrez-Quintana et al., 2022). However, excessive PARP-1 activation can lead to detrimental consequences, including neuronal death, persistent microglial activation, and neuroinflammation. The most well-established mechanism by which PARP-1 contributes to neuroinflammation involves its regulation of pro-inflammatory transcription factors such as NF-κB, AP-1, and nuclear factor of activated T cells (Ullrich et al., 2001; Kauppinen and Swanson, 2005; Kauppinen et al., 2011; Martínez-Zamudio and Ha, 2014; Stoica et al., 2014; Raghunatha et al., 2020) (Figure 1). In particular, several studies have reported that nuclear translocation of NF-κB requires PARP-1 function. NF-κB is one of the best-characterized transcription factors, regulating the expression of multiple genes involved in immunity and inflammation. PARP-1 activity is strongly linked to BBB disruption observed in neuroinflammatory diseases. While the precise mechanisms remain to be fully elucidated, several studies suggest connections between PARP activation, edema formation, and heightened infiltration of peripheral immune cells into the brain parenchyma (Chiarugi and Moskowitz, 2003). These observations led to the evaluation of PARP-1 inhibitors as potential mitigators of neurotoxicity in animal models of CNS pathologies in which neuroinflammation plays a key role. Moreover, of particular significance for their potential applications in neuro-oncology, PARP inhibitors have demonstrated synergistic effects when combined with DNA-damaging agents like TMZ and RT, which together constitute the standard of care for GBM patients (Lescot et al., 2010; Wu et al., 2014; Rom et al., 2015).

### 2.2 RT induces ER stress-response leading to neurodegeneration

Although the correlation between RT and neuroinflammation is largely been discussed, the precise mechanisms of neurotoxicity and consequent neurodegeneration remain poorly understood at the molecular level (Friedl et al., 2022). We tried to address this issue investigating the interaction between RT and ER-stress response pathways, building upon existing evidence linking RT to ER stress and ER stress to neurodegeneration. This approach allowed us to formulate a hypothesis about the specific link between RT and neurodegeneration.

Approximately one-third of all proteins undergo post-translational modifications, folding, and trafficking within the endoplasmic reticulum (ER) (Black et al., 1981; Kim et al., 2008) and cells maintain a state of proteostasis through a complex network of signaling pathways that regulate protein synthesis, folding, trafficking, and degradation (Read and Schröder, 2021). In response to specific physiological or pathological conditions, the demand for protein synthesis can surge, overwhelming the protein-folding capacity of the ER lumen. This results in the accumulation of partially folded, misfolded, or unfolded proteins, a state known as ER stress (Gutierrez-Quintana et al., 2022). Mild ER stress is typically managed by the unfolded protein response (UPR). However, prolonged or persistent ER stress triggers constitutive UPR activation, which ultimately leads to the activation of cell death pathways. Disruptions in these processes can lead to the accumulation and aggregation of misfolded proteins within cells, triggering pathological consequences, just as neurodegeneration (Ren et al., 2021).

In particular, radiation can induce ER stress either directly or indirectly through the production of reactive oxygen species (ROS). In some cases, cancer cell clones that survive radiation therapy may do so by activating compensatory survival signaling pathways, such as the UPR. In particular, under radiation-induced ER stress, specific signaling by PERK, ATF6, and IRE1 may be activated, and augment the upregulations of UPR-related genes to recover and recycle misfolded proteins (Chatterjee et al., 2018). However, excessive activation of UPR in the surviving cell fraction resident in the irradiated field, was showed to cause either radioresistance in tumor cells (Urra et al., 2016) and induce an anomalous accumulation of misfolded protein in neuronal cells exposed to radiation, paving the way to the pathogenesis of neurodegenerative disorders (Wang et al., 2018). Indeed, neuronal cells are particularly susceptible to protein misfolding compared to non-neuronal cells. In non-neuronal cells, cell division helps to mitigate the effects of ER stress by repeatedly diluting unfolded peptides. In contrast, not-dividing post-mitotic neurons rely solely on the UPR for survival. Therefore, if the misfolding is not resolved and normal cellular functions are not restored, the UPR can trigger selective neuronal death or neurodegeneration due to the accumulation of aberrant proteins. This strongly supports the crucial role of ER stress in the pathogenic neuronal response (Hetz and Saxena, 2017; Wang et al., 2018). Thus, various neurodegenerative diseases display specific types of misfolded proteins (Lindholm et al., 2006; Remondelli and Renna, 2017). For example, AD, PD, Huntington’s disease (HD), and ALS are characterized by a clinically silent period, during which aberrant proteins progressively aggregate and accumulate in the brain, leading to impaired synaptic function and ultimately neurodegeneration (Ciechanover and Kwon, 2015; Remondelli and Renna, 2017). These pathological conditions affecting the peripheral and CNS are also called “protein misfolding diseases.”

**Figure 2:**
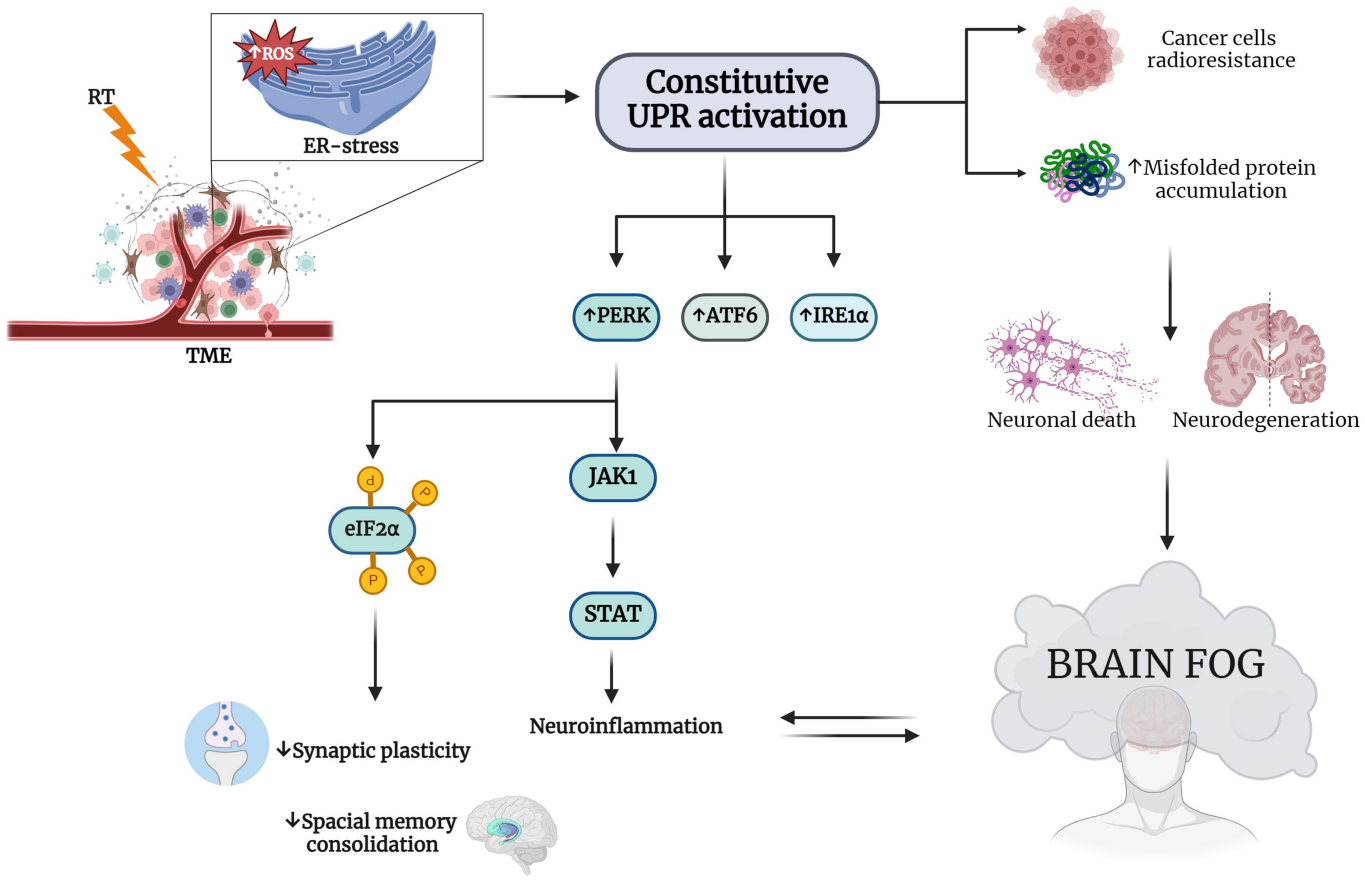
RT induces ER stress in TME and consequent brain fog. RT-induced endoplasmic reticulum (ER) stress-specific signaling that leads to a constitutive unfolded protein response (UPR) activation This increases the expression of eukaryotic initiation factor 2α (eIF2α) kinase (PERK), activating transcription factor 6 (ATF6) and type I transmembrane protein inositol requiring 1 α (IRE1α). Hyperactivation of UPR is a survival strategy from RT for cancer cells and results in misfolded protein accumulation in neuronal cells Chatterjee et al., 2018; Wang et al., 2018; Ren et al., 2021). The neurodegeneration induced by this accumulation may explain the cognitive impairment observed in patients treated with RT. PERK increasing determines on one hand the phosphorylation of eIF2α, decreasing synaptic plasticity and impairing spatial memory consolidation, and on the other hand, it causes neuroinflammation through Janus kinase 1 (JAK1) and the increasing of signal transducer and activator of transcription (STAT), with the consequent establishment of the so-called brain fog (Tang et al., 2001; van den Berg et al., 2001; Bellezza et al., 2014; Meares et al., 2014; Drake, 2015; Logsdon et al., 2016; Sprenkle et al., 2017). Created with BioRender.com.

In support of this, chronic ER dysfunction was showed to be highly associated with memory and cognitive impairment observed in different neurodegenerative diseases, like AD (Duran-Aniotz et al., 2014) and PD (Ryu et al., 2002; Colla et al., 2012). In addition, there is some evidence that PERK and IRE1, central in UPR signaling pathways, are important in neurodegenerative diseases due to their impact on synaptic functions and their capability to attenuate the effects of chronic ER stress. It has been shown that selectively lowering PERK expression in AD mice models prevents the aberrant phosphorylation of eIF2α and consequently improves synaptic plasticity and spatial memory consolidation (Costa-Mattioli et al., 2009) (Figure 2).

In addition, lowering of PERK expression in AD mice models prevent the aberrant phosphorylation of eIF2α and consequently improved synaptic plasticity and spatial memory consolidation (Costa-Mattioli et al., 2009). Moreover, suppression of eIF2α kinases alleviates AD-related plasticity and memory deficits (Ciechanover and Kwon, 2015). These findings, taken together, further support the hypothesis that UPR pathways are implicated in the disruption of cognitive and memory functionality and strategies aimed at restoring the proper proteostasis of neuronal cells could have important therapeutic effects (Hetz and Saxena, 2017). In fact, targeting pathways associated with abnormal ER stress with pharmacological treatment has been shown to rescue neuronal loss in PD *in vitro* models (Chung et al., 2013). There are also evidences that cells with a chronic and severe ER stress, for instance induced by RT, interfere with immunosuppressive environment of the CNS, supporting a link between neuronal cells under ER stress and glial cells leading to inflammation of brain microenvironment (Drake, 2015; Logsdon et al., 2016). As a case in point, ER-stress-induced astrocyte activation can induce a pro-inflammatory phenotype in microglial cells (Meares et al., 2014), which through their innate receptors, can recognize extracellular protein aggregates or oligomers as danger signals. This interaction triggers a neuroinflammatory response that initiates debris clearance via microglia-mediated phagocytosis (Sprenkle et al., 2017).

Notably, during the UPR the nuclear factor kappa-light-chain-enhancer of activated B cells (NF-kB) and mitogen-activated protein kinase 1 (MAPK-1) were shown to play a pivotal role in mediating cell survival (Tang et al., 2001; van den Berg et al., 2001). Furthermore, in astrocytes it has been shown that UPR affects STAT pathways through the interaction of PERK and JACK1, further supporting the association between UPR and neuroinflammation (Meares et al., 2014), paving the way to our hypothesis that RT could lead to neurodegeneration through the exacerbation of ER-stress response pathways.

### 2.3 RT effects on proteasomal degradation system and neurocognitive disorders

To counteract against misfolded proteins, the ER system has in place quality control mechanisms, including the unfolded protein response (UPR), as well as ER-associated degradation (ERAD) (Taylor et al., 2002; Hartl, 2017), which interacts in a coordinated manner with the ubiquitin-proteasome system (UPS) (Araki and Nagata, 2011). This multicatalytic complex is also the main target of many cancer therapies, including radiation. It was reported that even subtle changes in cellular redox balance caused by irradiation (and other stress stimuli) profoundly impact proteasome function. This suggests that proteasomes act as sophisticated and highly sensitive stress sensors, rapidly and simultaneously orchestrating diverse cellular processes in response to radiation exposure (Pervan et al., 2005). In particular, ionizing radiation exposure has been shown to impair normal proteasome activity. This reduction in proteasome activity slows the degradation of proteins, leading to their further accumulation and exacerbating endoplasmic reticulum (ER) stress conditions. Studies utilizing proteasome inhibitors across various organisms have revealed its impact on memory processes, including consolidation, recollection, and extinction. In fact, within the nervous system, the proteasome plays a crucial role in protein degradation and maintaining cellular homeostasis in neurons, glial cells, thereby contributing to overall brain health (Davidson and Pickering, 2023). Moreover, because the proteasome degrades most short-lived cellular proteins, primarily the proteasome subtypes (26S), changes in its activity might significantly, and selectively, alter the life span of many signaling proteins and in particular, in brain cells, compromise several neuronal functions, such as gene transcription and neurotransmitter release. Emerging research has shed light on the neuron-specific functions of the proteasome, particularly its crucial role in facilitating long-term memory formation (Giulivi et al., 1994; Dantuma and Lindsten, 2010; Brodsky and Skach, 2011; Jung and Grune, 2013) and potentiation (Pacifici et al., 1993), dendritic spine growth (Upadhya et al., 2004) and neurodevelopment (Dong et al., 2008; Hamilton et al., 2012), as well as synaptic plasticity (Hamilton and Zito, 2013). The proteasome also plays a regulatory role in clock proteins within the nervous system, influencing circadian rhythm (Ceriani et al., 1999).

**Figure 3:**
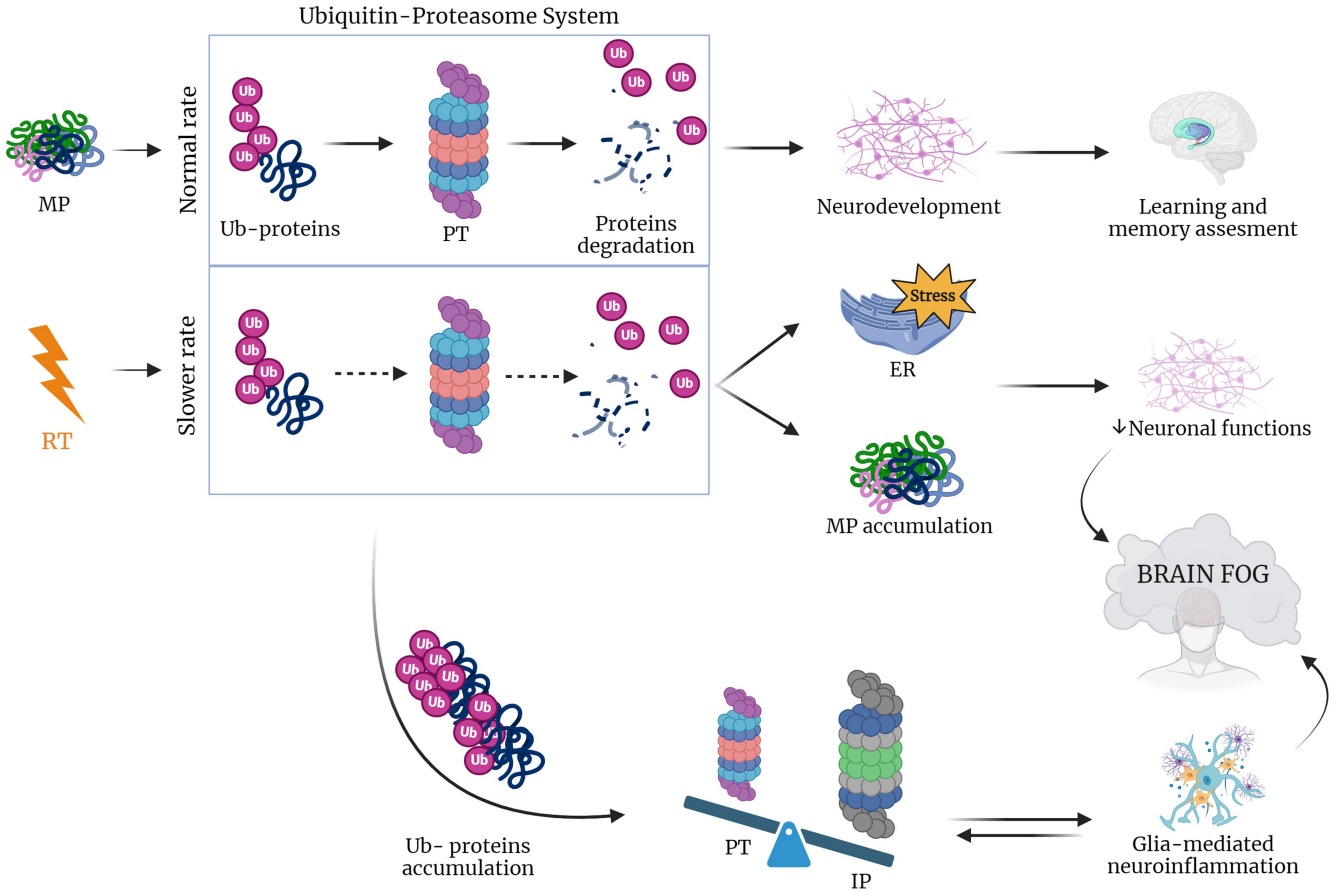
RT lowers UPR rate inducing brain fog. When protein misfolding occurs during physiological conditions proteins are ubiquitinated (Ub-proteins), activating the proteasome (PT) that in response induces protein degradation (Tang et al., 2001; van den Berg et al., 2001; Bellezza et al., 2014; Meares et al., 2014; Drake, 2015; Logsdon et al., 2016; Sprenkle et al., 2017). In the case of RT, this rate is lowered leading to the accumulation of Ub-proteins that shift the equilibrium to an increased impact of immunoproteasome, which leads to glia-mediated neuroinflammation. The latter, together with the accumulation of misfolded proteins and induced ER stress causes the compromise of neuronal function and the consequent brain fog establishment (Pearl-Yafe et al., 2003; Ferrington et al., 2008; Pintado et al., 2012; Orre et al., 2013; Jansen et al., 2014). Created with BioRender.com.

Furthermore, the proteasome is linked to neuroinflammation and to some age-related neurodegenerative diseases (Pintado et al., 2017). In particular, during neuroinflammatory conditions the brain expresses cyclooxygenases-1 and 2 (COX-1 and COX-2), which release prostaglandins that induce proteasome inhibition that, in turn, hampers neuroinflammation. In particular, COX-2 is upregulated in both neurons and glial cells during neuronal injury (Consilvio et al., 2004; Ishii et al., 2005; Bi et al., 2012). Additionally, in a study of Pintado and colleagues, it was shown that proteasome inhibition in a rat model caused a worsening of neuroinflammation (Pintado et al., 2012). Neurological disorders have been also reported to be associated with the accumulation of ubiquitinated proteins in neuronal inclusion and also with signs of inflammation that may contribute to neurodegenerative processes (Lev et al., 2006; Ortega et al., 2007). Taken together, these considerations lead to the hypothesis that changes induced by radiotherapy in proteasome system trigger the neuroinflammation which in turn induce cognitive impairment and neurodegeneration (Figure 3).

Declines in the activity of the constitutive proteasome, observed with aging and neurodegenerative diseases, often coincide with an upregulation of the alternative proteasome form, known as the immunoproteasome. The immunoproteasome is a specialized variant that differs from the standard proteasome in three subunits, induced by inflammation and constitutively expressed in hematopoietic cells. Under non-inflammatory conditions, the immunoproteasome is only a small portion of the total cellular proteasome pool, as immunoproteasome expression is low in neurons and glia in absence of cytokine stimulation. In the CNS the immunoproteasome appears to be expressed both in immune and non-immune cells, including in astrocytes, bone marrow-derived immune cells, oligodendrocytes, and Purkinje cells (Speese et al., 2003; Eide et al., 2005; Hegde, 2010). Immunoproteasome expression is typically low in these regions but undergoes a significant increase in response to injury (Ferrington et al., 2008). For instance, after interferon-γ release and during neuroinflammation, cells are stimulated to produce ROS such as the H_2_O_2_ and the superoxide hydroxyl radicals, thus damaging the cellular proteome (Pearl-Yafe et al., 2003). Moreover, when neuronal accumulation of ubiquitinated proteins occurs, there is a switch from proteasome to immunoproteasome that increases the peptide reserve for antigen presentation. Indeed, a Pintado’s *in vivo* study, showed that after the injection of lipopolisaccaride in rats with a higher proportion of immunoproteasome, proteasome inhibition induced the formation of neuronal aggresome-like structures. However, these modifications were not observed when proteasome inhibition was induced separately, suggesting that neuroinflammation is a crucial risk factor for intracellular protein accumulation and neurodegeneration (Pintado et al., 2012). Furthermore, the immunoproteasome plays a crucial role in glial cells, implying an interplay between the immunoproteasome and glia-mediated inflammatory responses, ultimately contributing to a pro-inflammatory environment (Orre et al., 2013; Jansen et al., 2014). Radiotherapy (RT) is known to trigger neuroinflammation, which in turn is associated with activation of the immunoproteasome. This activation has been linked to the formation of aggresome-like structures in neurons. Based on this chain of events, it’s conceivable that RT contributes to neurodegeneration and cognitive impairment in GBM patients by triggering of inflammatory response pathways and accumulation of misfolded proteins potentially damaging neurons in healthy tissue surrounding tumor.

### 2.4 RT’s CNS damage: a multifaceted attack

To recapitulate, RT triggers interconnected pathways, with neuroinflammation and glial activation as central players, ultimately leading to neuronal death, impaired communication, and cognitive decline known as brain fog. Understanding these pathways holds promise for developing neuroprotective strategies. The key pathways consist firstly in neuroinflammation: RT triggers inflammatory mediators (eATP, interferons, chemokines) & oxidative stress (IL-6, IL-10, TGF-β), leading to BBB disruption and further inflammation. This fuels neuronal death, memory issues, and glial activation (microgliosis & astrogliosis); secondly glial activation: microglial hyperactivity and astrogliosis release neurotoxic factors, damaging neurons and impairing communication; thirdly neurogenesis disruption: RT hinders new neuron formation in the hippocampus, impacting learning and adaptation; fourth DNA damage and senescence: DNA damage in astrocytes triggers a response leading to tissue damage and cognitive decline and fifth ER-stress: RT-induced the accumulation of misfolded proteins which activates UPR pathways, causing both radioresistance and neurodegeneration. Additionally, UPR interacts with JAK1, affecting STAT pathways and promoting neuroinflammation, that, in turn, stokes the vicious cycle that ends with brain fog (Table 1).

**Table 1:** RT’s CNS damage: a multifaceted attack.

RT-induced pathways leading to CNS damage	References
Release of inflammatory mediators (eATP, interferons, chemokines)	Greene-Schloesser et al. (2012), Herrera et al. (2017)
Increase of oxidative stress through the release of immunosuppressive molecules (IL-6, IL-10, TGF-β) with loss of BBB integrity	Alghamri et al. (2021)
Induction of reactive state of microglia that induces astrogliosis, release of neurotoxic factors, neuronal cell death, memory dysfunctions	Hwang et al. (2006), Wilson et al. (2009), Liddelow et al. (2017), Feng et al. (2018)
Perturbation in neurogenic niche leading to deficits in neural precursors, dysfunction in hippocampal neurogenesis, synaptic structure	Tada et al. (1999), Monje et al. (2003), Rola et al. (2004), Stevens et al. (2007)
DNA damage accumulation leading to a senescence-associated secretory phenotype in astrocytes with tissue damage and cognitive decline	Monje et al. (2002), Eriksson and Stigbrand (2010), Turnquist et al. (2019), Tripathi et al. (2021)
higher expression level of NF-kB and PARP 1 and consequent neuronal death, and constitutive microglia activation	Stoica et al. (2014), Kauppinen and Swanson (2005), Kauppinen et al. (2011), Raghunatha et al. (2020), Martínez-Zamudio and Ha (2014), Ullrich et al. (2001), Gutierrez-Quintana et al. (2022), Chiarugi and Moskowitz (2003)
Induction of ER-stress through the production of ROS, upregulation of UPR pathways (PERK, ATF6, IRE1) causing radioresistance through MAPK-1 and NF-kB and accumulation of misfolded proteins in neuronal cells	Tang et al. (2001), van den Berg et al. (2001), Drake (2015), Logsdon et al. (2016), Pintado et al. (2017), Chatterjee et al. (2018), Wang et al. (2018), Ren et al. (2021)
interaction between PERK and JACK1 affecting STAT pathways leading to neuroinflammation	Bellezza et al. (2014), Meares et al. (2014), Sprenkle et al. (2017)
Changes in cellular redox balance which impact on proteasome that act as a stress signal causing degradation of proteins and exacerbating ER-stress	Pacifici et al. (1993), Giulivi et al. (1994), Dantuma and Lindsten (2010), Davidson and Pickering (2023)
Induction of COX-1 and COX-2 upregulation with the release of prostaglandins that induce proteasome inhibition that hampers neuroinflammation contributing to neurodegeneration	Consilvio et al. (2004), Ishii et al. (2005), Bi et al. (2012)
Induction of neuroinflammation with release of IFN-γ, production of ROS that damage constitutive proteasome with the consequent upregulation of the immunoproteasome, which in turn induces the formation of neuronal aggresome-like structures and neurodegeneration	Pearl-Yafe et al. (2003), Ferrington et al. (2008), Pintado et al. (2012), Orre et al. (2013), Jansen et al. (2014)

## 3 New horizons for clearing brain fog

Due to their highly invasive nature and extensive infiltration of brain tissue, GBM treatment often involves delivering high doses of RT (typically 60 Gy) to large brain volumes in an attempt to delay tumor recurrence and extend patient survival. However, this unavoidably exposes normal, functioning brain tissue to radiation, that causes devastating effects on brain function. Radiation-induced cognitive impairment manifests with acute (days to weeks after RT), early delayed (1–6 months after RT and often reversible), and late delayed effects (6 months or more after RT and usually irreversible and progressive). Late delayed effects include decreases in memory and executive functioning, among other deficits, further worsening the quality of life of GBM patients. The mechanisms underlying RT-induced neurotoxicity are still being studied and are known to be complex and multifaceted. This complexity makes it challenging to develop effective preventive measures to mitigate the adverse effects of RT on the brain. In the present review, we have focused on the molecular mechanisms that have been found to play a central role both in the etiology and pathogenesis of cognitive impairment due to RT and to important degenerative diseases, such as AD and PD.

About this, the sigma receptors (SRs), a class of ER transmembrane proteins, could represent an appealing target for the prevention of neurocognitive disorders. Sigma receptors (SRs) exist in two subtypes: sigma-1 receptor (S1R) and sigma-2 receptor (S2R). S1R resides on the mitochondria-associated endoplasmic reticulum (ER) membrane (MAM), while S2R is found in the ER-resident membrane. SRs exert chaperoning functions and modulate physio-pathological processes in the CNS. SRs are found abundantly in various brain cells, including neurons, astrocytes, microglia, and oligodendrocytes (Gundlach et al., 1986; Alonso et al., 2000; Hayashi and Su, 2004; Gekker et al., 2006; Zhao et al., 2014).

A multitude of studies have demonstrated that S1R plays a crucial role in promoting neuronal survival and restoring neuronal functions in neurodegenerative diseases. This neuroprotective effect is attributed to S1R’s ability to modulate various cellular processes (including calcium homeostasis and glutamate activity), reducing the production of ROS, regulating ER and mitochondrial functions, and influencing reactive gliosis and neuronal plasticity (Nguyen et al., 2015; Ruscher and Wieloch, 2015). Emerging research has demonstrated the effectiveness of S1R-targeting drugs in alleviating symptoms associated with a wide range of neurodegenerative disorders, each with distinct underlying mechanisms. These disorders include learning and memory disorders, cognitive impairments, and neurodegenerative diseases such as AD, PD, ALS, MS, and HD (Maurice and Goguadze, 2017).

In addition to S1R, S2R has also been shown to play an important role in neurological diseases (Huang et al., 2014). S2R couples and interacts with surrounding proteins to actuate a wide variety of cellular processes being closely associated and interacting with key proteins including progesterone receptor membrane component 1 (PGRMC1). S2R and PGRMC1 are linked to learning and memory through mechanism of action studies and efficacy studies in *in vitro* and *in vivo* preclinical models. PGRMC1 is also a well-identified hormone receptor with multiple functions in AD (Xu et al., 2022), and α-synucleinopathies (Kline et al., 2017). Indeed, S2R modulators have been shown to ameliorate amyloid-β oligomer and α-synuclein oligomer-mediated deficits in neuronal trafficking (Izzo et al., 2014).

Based on the many pathways affected in neurodegenerative diseases, another possible good candidate could be the hyperbaric oxygen treatment (HBOT). This therapy has been used for over 50 years to treat various conditions, including decompression sickness and wound healing (Mensah-Kane and Sumien, 2023). Recent studies have shown promising results in using HBOT to treat conditions associated with neurodegeneration and functional impairments. In fact, HBOT has been shown to reduce neuroinflammation in severe brain disorders. It also has the ability to downregulate pro-inflammatory cytokines (IL-1β, IL-12, TNFα, and IFNγ) while upregulating an anti-inflammatory cytokine (IL-10), making it potentially cytoprotective (Kudchodkar et al., 2008). Moreover, combining hyperbaric oxygen (HBO) with RT was found to suppress inflammasome activation in an *in vitro* human microglia model (Arienti et al., 2021). This effect was attributed to a reduction in the expression levels of pro-inflammatory cytokines IL-1β and IL-6. Similar results were obtained by Qian et al. who reported that, in animal models, HBO mitigates the inflammatory response associated with traumatic brain injury by modulating microglial inflammasome signaling (Qian et al., 2017). Recently, basic and clinical research has shown the potential of HBOT to treat neurodegenerative diseases (Huang and Obenaus, 2011; Huang et al., 2016; Shapira et al., 2018). The effectiveness of HBOT in improving age-related cognitive decline was evaluated in a study involving healthy elderly individuals (Jacobs et al., 1969). Male participants with an average age of 68 years, displaying clinical signs of intellectual deterioration, underwent cognitive assessments following 30 intermittent sessions of HBOT, which involved breathing pure oxygen at 2.5 times atmospheric pressure. HBOT enhanced cognitive function in these healthy older adults through mechanisms involving regional alterations in cerebral blood flow, as assessed by perfusion magnetic resonance imaging (Amir et al., 2020). Also, elderly patients with significant memory loss demonstrated enhanced cognition and increased cerebral blood flow following exposure to HBOT (Shapira et al., 2018). In the *in vivo* models of aging, HBOT effectively counteracted cognitive decline and hippocampal-dependent pathologies by enhancing cholinergic signaling pathways, protecting against apoptosis, and mitigating oxidative stress and inflammatory responses (Chen et al., 2016, 2017; Shwe et al., 2021).

Another common mental disease associated with brain tumors and RT is depression. The impact of stress on brain morphology has become increasingly evident through extensive research spanning several decades and depression is now clearly associated to chronic uncontrollable stress and the related neuroinflammation derived (Eyre and Baune, 2012; Iwata et al., 2013; Franco and Fernández-Suárez, 2015). Indeed, depressed patients usually exhibit increased inflammatory cytokines such as IL-1β, IL-6 and IFNγ, both in different brain regions and the periphery (Maes et al., 2009; Wager-Smith and Markou, 2011; Young et al., 2014). It is also known that enhanced levels of IL1β in the hippocampus lead to inflammation, that may contribute to depression (Kovacs et al., 2016). In this context, the role of ATP-gated transmembrane cation channel P2X7 receptor in the neuroinflammation is highlighted, due to its involvement in the IL-1β maturation (Potucek et al., 2006; Mingam et al., 2008; Piccini et al., 2008). These receptors are mainly located on microglia and activated in response to stress signals like increased level of ATP (Ferrari et al., 2006). Studies on peripheral immune cells demonstrated that activation of P2X7R induced oligomerization of NLR family pyrin domain containing 3 (NLRP3) with other proteins that in complex form the so-called inflammasome (Yue et al., 2017). The latter, as already discussed, is associated to neuroinflammation, leading to the neurodegeneration that can comprehend, in the light of the last discoveries, depression. Taken together, these findings may suggest NLRP3 inflammasome as a new therapeutic target for cognitive impairment related to radiation therapy (Alcocer-Gómez and Cordero, 2014).

**Figure 4:**
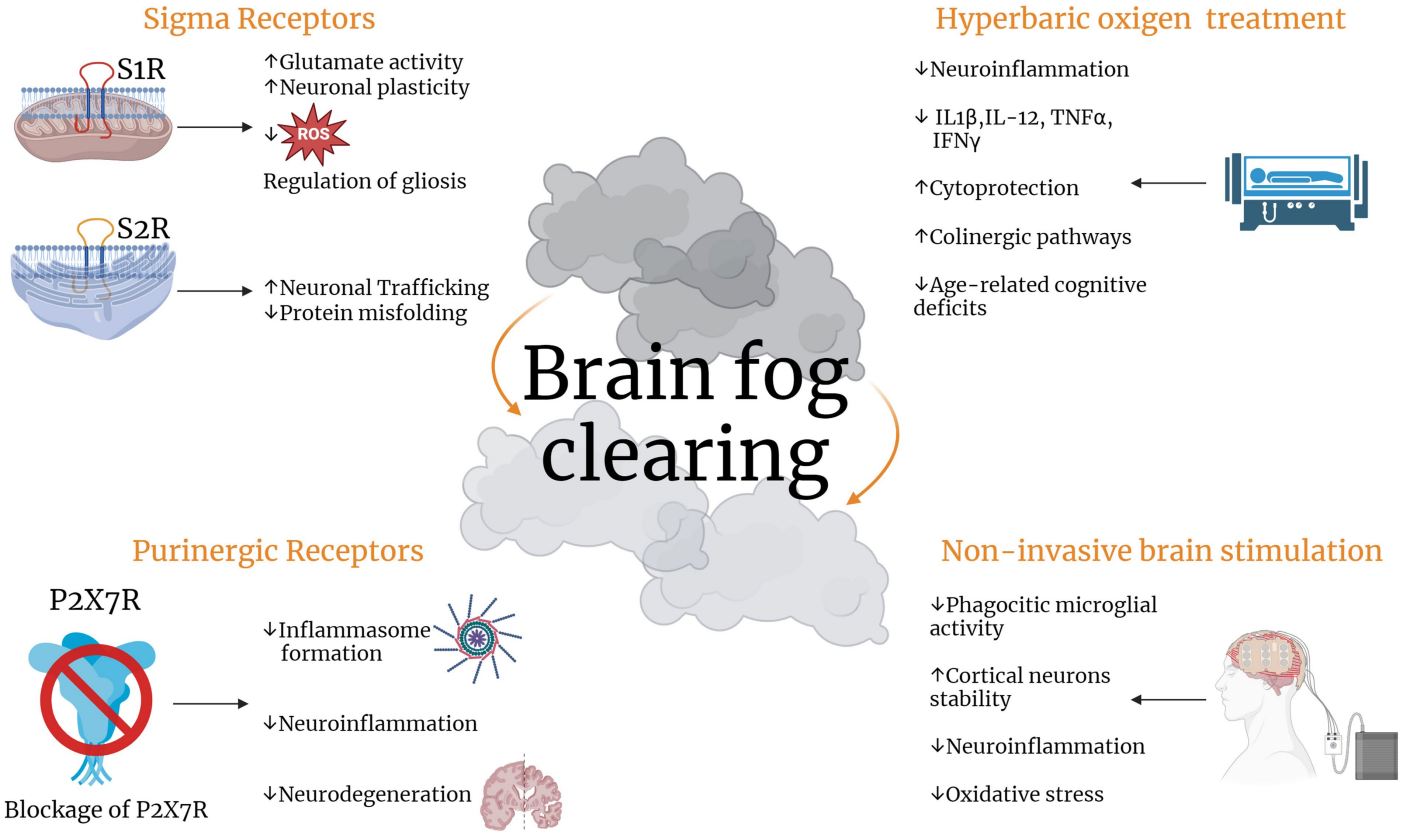
New therapeutic strategies to clear brain fog. Sigma receptors are ER transmembrane proteins represented by Sigma-1 receptor (S1R) and Sigma-2 receptor (S2R), that exert chaperoning functions and modulate physio-pathological processes like neuronal plasticity, glutamate activity as well as regulation glial cells reactivity. S1R receptor agonists preserve neurological functions while S2R antagonists ameliorate neuronal trafficking deficits derived by protein misfolding diseases. Hyperbaric oxygen treatment is involved in many neurodegenerative diseases. It reduces neuroinflammation through the inhibition of pro-inflammatory cytokines and mediate cytoprotection via the upregulation of anti-inflammatory cytokines. It also prevents cognitive impairments and hippocampal pathologies through the modulation of cholinergic pathways. ATP-gated transmembrane purinergic P2X7 receptor induces the formation of inflammasome by leading to oligomerization of NLRP3, posing the way to think at these receptors as a new therapeutic strategy to target cognitive impairment related to radiotherapy. Non-invasive brain stimulation techniques may add to the previously described agents and represent a new horizon on the brain fog treatment. They induce neuronal plasticity changes, reduce phagocytic microglial activity and neuroinflammation, and decrease oxidative stress while increasing cortical neurons stability. These could be innovative therapeutic target to clear brain fog. Created with BioRender.com.

Non-invasive brain stimulation (NIBS) may represent a new age of brain fog treatment. Neuromodulatory techniques stand as robust alternatives to pharmacological interventions for neurological and neuropsychiatric disorders, primarily due to their numerous advantages, including non-invasiveness, enhanced safety, and minimal to negligible side effects (Peruzzotti-Jametti et al., 2013a). Though NIBS tools developed on magnetic and electric fields, and more recently on ultrasound, resulting in one of the fastest-growing fields in medicine, the concept of influencing the activity of the human brain by using external therapeutical strategies dates back to the 1st century AD (Cambiaghi and Sconocchia, 2018). The main non-invasive brain neuromodulatory approaches are repetitive transcranial magnetic stimulation (rTMS), transcranial direct current stimulation (tDCS), transcranial alternating current stimulation (tACS), random noise stimulation (RNS), transcranial ultrasound stimulation (TUS). Although the underlying mechanisms of action is slightly different among them, NIBS tools are known to induce long-lasting neuronal plasticity changes, associated to behavioral modifications in both humans (Zhao and Woodman, 2021; George et al., 2022) and animal models (Cambiaghi et al., 2020a; Cherchi et al., 2022). Interestingly, in addition to neuronal effects, both magnetic and electric stimulation after effects have been recently associated with different glial cell activity modulation. Of note, in ischemic mouse models rTMS promotes microglia anti-inflammatory cytokines production both *in-vitro* and *in-vivo* (Luo et al., 2022). In rodent models of brain ischemia and vascular dementia, tDCS lead to an attenuation of the inflammatory response in different brain regions. In particular, in the MCAO mouse model of brain ischemia, cathodal tDCS is able to preserve cortical neurons if applied in the acute phase (Peruzzotti-Jametti et al., 2013b), while it exerts positive effects on functional motor outcomes when delivered hours after the brain damage and inflammatory response, combined with a less phagocytic anti-inflammatory microglia activity (Cherchi et al., 2022). In the rat vascular dementia model, anodal tDCS reduces the levels of malondialdehyd and ROS, but enhances superoxide and glutathione, thus reducing the oxidative stress (Guo et al., 2020). Finally, mice exposed to a 14-days 5 Hz rTMS exhibit increased cell proliferation in the hippocampal dentate gyrus, in parallel with improved cognitive behavior (Ramírez-Rodríguez et al., 2022). In line with this, 5 days of high-frequency (15 Hz) rTMS showed an improved emotional behavior paralleled by enhanced prefrontal cortex morphological plasticity, both in terms of dendritic spine density and dendritic complexity of layers II/III and V (Cambiaghi et al., 2022). On the contrary, 1 Hz rTMS results in augmented mature granule cells and newly generated neurons structural complexity, in association to antidepressant effects, though not affecting neurogenesis (Cambiaghi et al., 2020b). Especially, these latter observations on NIBS effects on glial cells, inflammation and neurogenesis well suits a strong interest for the treatment of brain fog associated to RT (Figure 4).

## 4 Conclusion

Radiotherapy (RT) is a common treatment for glioblastoma multiforme (GBM), but it can have adverse neurocognitive effects. The exact impact of RT on the quality of life in long-term GBM survivors is not fully understood. Predicting the clinical impact of RT is challenging because both the immediate and long-term effects of RT on quality of patient’s life depend on various factors. These factors include radiobiological factors (RT dose, volume, timing, and duration), physiological factors (pre-existing brain function), and patient-related factors (age, sex, and comorbidities). While RT remains the most effective non-surgical treatment option for GBM, its effectiveness is limited by the inherent and adaptive radioresistance of these tumors, which contributes to their inevitable recurrence.

Radiation treatment planning should consider the brain’s remarkable ability to adapt and recover by creating new neural connections, a crucial aspect of patient rehabilitation, as well as the sensitivity of the targeted brain regions. Even if the most severe effects occur months to years after radiation therapy, it is conceivable that decreasing the early impairment of brain parenchyma could likely prevent the propagation of the late-term effects of RT (Constanzo et al., 2020).

While our grasp of the underlying mechanisms of radiation-induced cognitive dysfunction remains incomplete, compelling evidence points to neuroinflammation as a significant contributor. Recent research has unveiled neuroinflammation as a pervasive feature in numerous CNS disorders, encompassing brain trauma, stroke, and various neurodegenerative processes. Bridging the gap between these preclinical findings and clinical practice holds the potential to enhance both survival rates and quality of life for brain tumor patients undergoing RT. Incorporating neuroinflammatory markers, cognitive function assessments, and quality of life measures into the design of future clinical trials based on RT treatment is crucial. Furthermore, literature data suggest a plausible link also between radiation neurotoxicity and UPR activation. However, targeting the UPR is still challenging, due to its role in physiological pathways that involve different organs, so it can have serious adverse effects if administered for a long time. Indeed, particular attention should be paid as the consequences on basal motor and cognitive functions could be severe and this aspect has to be taken into account (Hetz and Saxena, 2017).

## Author contributions

NM: Conceptualization, Writing – original draft, Writing – review & editing. MB: Writing – review & editing. MEV: Writing – original draft, Writing – review & editing. MC: Writing – review & editing. AT: Conceptualization, Writing – original draft, Writing – review & editing.
